# Mindfulness, Acceptance and Defusion Strategies in Smokers: a Systematic Review of Laboratory Studies

**DOI:** 10.1007/s12671-017-0767-1

**Published:** 2017-07-17

**Authors:** Shirley Serfaty, Grace Gale, Matthew Beadman, Brett Froeliger, Sunjeev K Kamboj

**Affiliations:** 1 Research Department of Clinical, Educational and Health Psychology, University College London, London, UK; 2 Department of Neuroscience, Medical University of South Carolina, Charleston, SC USA

**Keywords:** Mindfulness, Acceptance, Defusion, Emotion regulation, Supression, Reappraisal, Smoking, Smoking cessation, Analogue studies, Acceptance and Commitment Therapy, Craving, Negative affect

## Abstract

The psychological flexibility model (PFM) provides a framework for understanding and treating behavioural dysregulation in addictions. Rather than modulating the intensity of subjective experience, interventions based on, or consistent with, the PFM (PFM interventions) seek to alter the individual’s relationship to internal states, such as craving, negative affect and drug-related thoughts, using mindfulness, acceptance and related strategies. Experimental (non-clinical) studies in smokers have examined the effects of specific isolated strategies informed by or consistent with the PFM (PFM strategies). Here, we systematically review these studies and determine the extent to which they conform to methodological standards indicative of high levels of internal validity. Eligible studies were identified through electronic database searches and assessed for the presence of specific methodological features. Provisional aggregate effect sizes were determined depending on availability of data. Of 1499 screened publications, 12 met the criteria. All examined aspects of private subjective experience relevant to abstinence (craving *n* = 12; negative affect *n* = 10), demonstrating effects favouring PFM strategies relative to inactive control conditions. However, only six assessed outcome domains consistent with the PFM and provided no consistent evidence favouring PFM strategies. Overall, most studies had methodological limitations. As such, high-quality experimental studies continue to be needed to improve our understanding of necessary and/or sufficient constituents of PFM-guided smoking cessation interventions. Recommendations for future research are discussed.

## Introduction

It is a truism that most smokers want to quit and that most who make a quit attempt relapse even if they receive the best available psychosocial and/or pharmacological treatments (Fiore et al. 2008). As such, there is considerable scope for improving the efficacy of interventions for quitting smokers. This might involve relatively minor modifications to existing treatment approaches or a more radical shift in the way smokers are routinely helped to quit (Kamboj and Das 2017).

Existing behavioural interventions often encourage smokers to reduce exposure to relapsogenic situations or cues and/or promote the regulation of affect (including craving) through the use of strategies intended to modify the content, frequency or intensity of private psychological experiences. For example, smokers learn to identify and systematically dispute unhelpful thoughts (e.g. ‘one cigarette won’t hurt’). These strategies belong to a family of behavioural therapies—variously termed behavioural counselling, coping skills training or cognitive behavioural therapy (CBT)—that generally involve antecedent-focused emotion regulation strategies (which modulate emotions at input) like reappraisal or situation modification (e.g. avoiding places where smoking is likely). A primary assumption of these approaches is that regulation of (reduction in) craving and/or negative affect is required before behaviour change occurs.

However, some researchers have pointed to limitations of such cognitive-behavioural emotion regulation strategies. For example, the ubiquity of smoking cues, their frequent occurrence in both the external and the less easily regulated internal (bodily) environments, makes it impossible to avoid them. Moreover, attempts to apply certain response-focused emotion regulation strategies (which are deployed once an emotional response has commenced, e.g. suppression), to dampen craving, negative affect or smoking-related thoughts, can result in rebound effects (Salkovskis and Reynolds 1994; Sayers and Sayette 2013; Toll et al. 2001). More generally, strategies that emphasise control over internal experiences (thoughts and feelings) are likely only to be successful in those with significant pre-existing cognitive reserves, although even these individuals are susceptible to the ironic effects of over-control, especially under conditions of stress (Garland et al. 2014; Muraven and Baumeister 2000).

In contrast, advocates of recently developed smoking cessation interventions informed by contextual behavioural science consider efforts to downregulate private subjective experiences to be unrealistic, offering only temporary relief from unwanted experiences and generating an unworkable problem-solving mindset towards such experiences (Blackledge and Hayes 2001). They suggest an alternative approach to understanding addiction that is gaining influence, namely the psychological flexibility model (PFM), the principles of which are embodied and applied in Acceptance and Commitment Therapy (ACT; Hayes et al. 2011). The most relevant psychological constructs in ACT and other PFM-consistent interventions are self-regulated attention and openness or orientation to experience, characterised by acceptance of, and curiosity towards, current experience (Bishop et al. 2004). Interested readers are referred to authoritative texts on the PFM, which describe its underlying theory and therapeutic components in detail (e.g. Hayes et al. 1999).

Unlike CBT, which assumes that thoughts have some inherent truth value that can be disputed, the PFM proposes that cognitive disputation strategies give too much weight to thoughts and can promote maladaptive responding (cognitive fusion). Alternatively, therapeutic strategies that encourage an experiential understanding of the essentially illusory and transient nature of thoughts and feelings are foundational techniques in ACT and mindfulness-based addiction therapies (Hayes et al. 2011). In essence, changing the way individuals relate to their internal experiences (i.e. observing them with curiosity and accepting them as temporary mental processes that are not inherent to the self) can have the effect of discouraging experiential avoidance strategies, which are considered primary transdiagnostic maintaining factors in psychological/substance use disorders.

Whilst regulation of the intensity or occurrence of private psychological experiences is not the focus of PFM interventions, reductions in craving and negative affect may nonetheless occur indirectly and secondarily to increased attention towards (or reduced avoidance of) such experiences (Farb et al. 2014; Witkiewitz et al. 2013). This could occur, for example, through enhanced extinction of conditioned craving responses, during which simultaneous exposure to, and attention towards, multiple conditioned drug stimuli (in the absence of drug reward) could drive greater inhibitory learning (e.g. Treanor 2011). In contrast, ‘ordinary’ extended cue exposure has limited effects on smoking-related cue reactivity (subjective craving and physiological responding), even when combined with drugs that ostensibly enhance extinction learning (e.g. Kamboj et al. 2012). Alternatively, mindfulness-like strategies could interrupt (compete with) the elaboration of desire-related thoughts, with knock-on effects on craving (Farb et al. 2014; May et al. 2011). To reiterate, however, direct attempts to moderate the intensity of internal experiences are not employed in PFM-guided interventions and would not feature in the rationale for their use as presented to treatment recipients. Indeed, given the paradoxical nature of instructions to approach and ‘stay with’ sensations of craving or negative affect, provision of a clear rationale and, preferably, experiential exercises to exemplify the purpose of these strategies would seem to be essential to ensuring compliance with techniques that may, at first blush, be experienced as counterintuitive (Levin et al. 2012).

ACT is a multi-component treatment. Four of the components of ACT—contact with the present moment, acceptance, defusion and self-as-context—fall under the umbrella of mindfulness and acceptance practices (values and committed action are its fifth and sixth elements; Hayes et al. 2011). In contrast, mindfulness-based relapse prevention (MBRP) is monomodal insofar as the central therapeutic activities are various mindfulness meditation exercises (Bowen et al. 2014). Although mindfulness is conceptualised somewhat differently in ACT than in MBRP and related mindfulness treatments, we argue that there is sufficient convergence between these approaches (in terms of inculcating a particular attitude to the here and now and in aspects of the definition of mindfulness) to consider their constituents to belong to the same family of PFM-consistent strategies (or, simply, PFM strategies).

Evidence for efficacy of comprehensive PFM interventions for smoking cessation is accumulating. However, clinical trials are not ideally suited to testing the theoretical bases of the PFM. Rather, experimental laboratory studies that examine isolated components of these interventions can be more useful in this regard. Moreover, such lab-based studies allow the safety and relative efficacy of treatment components to be efficiently and cost-effectively tested. In this way, the most promising components can be retained and combined into optimised treatment packages and/or tested in more complex factorial designs (Hayes et al. 2013). Recently, Levin et al. (2012) reviewed laboratory studies that examined the effects of isolated components of the PFM, providing support for the efficacy of individual components across a broad range of participants and problem types. However, at the time of that review, few relevant studies had been conducted in smokers. Although previous studies have reviewed mind-body- and mindfulness-based treatments for smoking cessation either systematically (Carim-Todd et al. 2013; de Souza et al. 2015) or narratively (McCallion and Zvolensky 2015), no review that we are aware of has specifically examined the variety of components of the model and focused specifically on experimental component studies.

We systematically review laboratory studies of PFM strategies relevant to smoking cessation. Whilst we examine outcomes with established relevance to smoking cessation—specifically craving and negative affect—the more relevant outcomes from the perspective of the PFM include smokers’ relationship to these experiences (Levin et al. 2012), cessation-relevant values and, importantly, cessation-relevant behaviours. Whilst other psychosocial therapies would obviously also view a reduction in smoking as the primary goal of treatment, as noted above, behaviour change in these approaches is expected to be mediated by changes in negative affect or craving and/or smoking-related beliefs. In contrast, the PFM predicts a reduction in smoking independently of reductions in craving or negative affect (Brewer et al. 2013; Elwafi et al. 2013). Given these distinct goals of PFM strategies on one hand, and emotion regulation-based cognitive/behavioural strategies on the other, it should be clear that distinct outcome measures that tap these differing goals should ideally be used in experimental studies that assess these respective approaches. Specifically, whereas craving and negative affect are likely to be important outcomes in assessing emotion regulation-based approaches, measures of meta-cognitive/affective processes, values and/or behaviour change are more relevant to studies of PFM strategies (Levin et al. 2012). It was therefore of interest to determine the extent to which the most suitable outcome measures were routinely used in studies of PFM strategies in smokers. As craving, negative affect and smoking behaviour were the most commonly assessed constructs in the identified studies, provisional aggregate effect sizes are reported for these when data was (made) available.

## Method

The Preferred Reporting Items for Systematic Reviews and Meta-Analyses (PRISMA) were used for this review (Moher et al. 2009).

Eligible studies were required to be published in English language, peer-reviewed journals since 1987 (when aspects of the PFM were elaborated by Hayes 1987) and to test a recognised *component of* the PFM in adult smokers (≥18 years old). It was required that studies provided strategy instructions within an experimental session and that participants were assigned randomly or quasi-randomly to PFM and comparison strategies. Following Levin and colleagues (Levin et al. 2012), comparison conditions were classified as ‘active controls’ (involving recognised adaptive strategies that aim to modify the content, frequency or intensity of internal experience, e.g. cognitive reappraisal, as used in CBT), ‘inactive controls’ (strategies that lacked any theoretical or therapeutic basis relevant to smoking cessation but controlling for time and/or attention, e.g. reading) or ‘suppression’ (aiming to directly reduce or eliminate some aspect of internal experience). Included studies needed to report smoking-relevant meta-cognitive/affective outcomes and/or smoking behaviour (PFM-relevant outcomes) and/or outcomes relating to the occurrence, frequency and/or intensity of craving and/or negative affect.

We reiterate that the purpose of this review is to understand the contribution, based on experimental laboratory studies of specific isolated components of treatments used in comprehensive interventions. As such, studies reporting clinical trials of comprehensive treatments were excluded. Relatedly, to ensure that relevant effects were likely attributable to isolated, specific and well-defined (hence time-limited) PFM strategies, rather than non-specific elements within experiments, studies examining the effects of within-session strategy practice which likely lasted >1 h were also excluded. This arbitrary cutoff was chosen for convenience, and because it is likely to represent an upper limit of what meditation-naïve participants would be able to engage with in a single experimental procedure. We also excluded studies primarily reporting qualitative data, or examining the influence of mediating or moderating variables based on data from a previous study. Finally, studies were excluded if there was no mention of the theoretical basis or therapeutic objectives of PFM interventions. As such, studies that examined values in the context of ‘ego threat’—that is, studies of self-affirmation—were not included (cf Levin et al. 2012).

### Systematic Literature Search

An initial search of Embase, Medline and PsychINFO databases was conducted in November 2013 and updated in July 2016. The following search terms were used: [Title/abstract]: Acceptance and commitment therapy OR Accept* OR Defusion OR Present Moment OR Values OR Value directed behavio?r OR Self as context OR Commit* OR Psychological flexibility OR Mindful* OR Distress tolerance OR Relational frame* OR Behavio?ral OR Contextual Behavio?ral OR Third wave OR Metacognitive OR Meta-cognitive AND Smok* OR Nicotine* OR Tobacco* OR Cigarette?* OR Cessation OR Crav* OR Negative affect*.

**Fig. 1:**
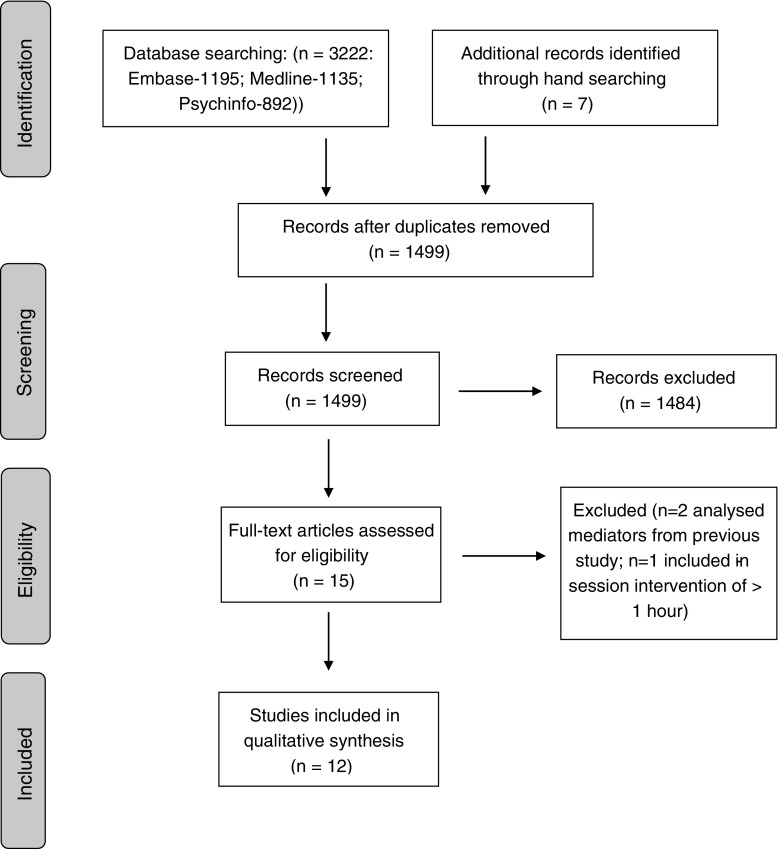
PRISMA flowchart

Searches yielded 3222 hits (1195 from Embase, 892 from PsychINFO, 1135 from Medline; see Fig. 1). Duplicates were removed and titles and/or abstracts of remaining studies (*n* = 1499) were screened for relevance. Full-text articles were obtained for studies that appeared to be eligible and additional hand-searching was conducted. Twelve papers met inclusion criteria (Beadman et al. 2015; Bowen and Marlatt 2009; Cropley et al. 2007; Litvin et al. 2012; May et al. 2011; Nosen and Woody 2013; Rogojanski et al. 2011; Ruscio et al. 2016; Szasz et al. 2012; Ussher et al. 2009; Ussher et al. 2006; Westbrook et al. 2013).

### Methodological Assessment of Studies

Each study was assessed against criteria recommended by Barnes-Holmes and Hayes (2003), describing optimal features of PFM component studies. To facilitate this—especially to determine the level of matching between conditions (item 3 below)—authors of studies were contacted and asked to provide strategy instructions if these were not available in published reports. These were available for all but one study (Nosen and Woody 2013). Studies and strategy instructions were assessed by the first author, and a random sample of six studies were evaluated independently by the senior author. Assessment involved determining the presence (or likely presence) of the specific methodological features (14 in total) outlined below on a ‘yes’ (study feature present) or ‘no’ (study feature absent) scale. Where assessments diverged, agreement was reached through discussion between assessors. It should be noted that we do not make any claims about the psychometric properties of this rating scale. It was not intended to provide a valid quality rating of studies but rather to highlight the presence or absence of certain recommended study features for PFM component studies. The following methodological features were assessed: (1) blinding of experimenters to condition; (2) presence of pre-existing group differences and use of statistical methods to examine/control for these if appropriate; (3) matching of strategy instructions in different experimental conditions for length, complexity (assessed here for individual instruction sets using the Flesch-Kincaid readability level), number of smoking-relevant words and engagement with the material and delivery method; (4) appraisal of features in criterion 3 by independent raters; (5) direct relevance and connection of strategies to craving and negative affect (e.g. mindfulness directed at craving or negative affect); (6) checking of theme/quality of strategies and their relevance to the experimental challenge by independent raters (e.g. mindfulness instructions actually included mindfulness constructs that related to craving); (7) verbal articulation of understanding of strategies by participants; (8) if appropriate, use of a reminder to use the appropriate strategy prior to physical/psychological challenge; (9) use of standardised instructions; (10) if relevant, summaries of the actual strategy used by participants are provided by participants and independently checked; note that this contrasts with criterion 7, which relates to checking comprehension (prior to application of the strategy); (11) credibility/expectancy checks obtained; (12) objective manipulation checks performed assessing extent of comprehension and application of strategies; (13) adequate power (studies with *n* ≥25 per condition—or a sample size based on an a priori power calculation—were considered to be adequately powered based on effect sizes reported in Beadman et al. (2015) and Szasz et al. (2012)) and (14) strategy involves an active/experiential component rather than a rationale alone.

### Effect Sizes

Depending on reporting or provision of means and SDs upon request from authors, weighted mean effect sizes (ESs; standardised mean differences) were determined using Review Manager 5.3 software according to the equation outlined in Deeks and Higgins (2010), using random-effect models (Cumming 2013). Only when at least 75% of means/SDs for a particular outcome/comparison were available was an aggregate ES reported. It is acknowledged, however, that non-availability of data may have biased the reported aggregate ESs.

## Results

### Participant Characteristics

Participants (*n* = 952; 53.88% men) had an average age of 32.67 years and a range of baseline smoking-related characteristics. Six studies (Beadman et al. 2015; Bowen and Marlatt 2009; Litvin et al. 2012; Rogojanski et al. 2011; Szasz et al. 2012; Westbrook et al. 2013) specifically recruited participants with some intention or desire to quit smoking. One study provided no information on levels of dependence (May et al. 2011), but the others generally indicated that participants were mild to moderately dependent, with a mean baseline Fagerström Test for Nicotine Dependence (FTND) score of 4.35 (*n* = 9 studies). Two studies that used either the Cigarette Dependence Scale (Etter 2008) or the Wisconsin Inventory of Smoking Dependence Motives (Piper et al. 2008) also reported mean scores indicative of mild to moderate dependence (Nosen and Woody 2013; Ruscio et al. 2016). One exception to this was the Bowen and Marlatt’s (2009) study, which was an outlier with notably lower (~2 SDs) FTND scores, suggesting that their sample was distinct from the smokers in other studies reviewed here. Their participants were also the youngest (see Table 1).

**Table 1:** Study characteristics

Study	Number	Experimental conditions	Baseline sample characteristics (mean except where indicated)	Follow-up details (instructions for use of strategy; percent retention)	Behavioural and other PFM-consistent outcomes	Outcomes relating to the frequency and/or intensity of internal experiences	Credibility/manipulation check
1. Beadman et al. (2015)	73	Experimental: defusion. Written instructions: 813 words including cue reactivity instructions Suppression control: written instructions: 810 words including cue reactivity instructions Active control: reappraisal (written instructions: 791 words including cue reactivity instructions)	Age: 24.87 Male: 51.0% FTND: 5.14 Cigs/day: 13.00 Abstinence: 5.61 h	Explicit instructions to continue to use assigned strategy 24 h (90.41%) 7 days (71.23%; imputation of missing values)	Compared suppression Latency to smoke ↑ ^Post-session^ Number of cigarettes ↓^7 days^ Experiential avoidance ↓ ^In session^ Compared to reappraisal Latency to smoke *↔* ^Post-session^ Number of cigarettes (TLFB)*↔* ^7 days^	Compared to suppression Craving (QSU brief) *↔* ^In session, 24 h, 7 days^ Negative affect (PANAS) *↔* ^In session^ Compared to reappraisal Craving (QSU brief) *↔* ^In session, 24 h, 7 days^ Negative affect (PANAS) *↔* ^In session^	Written descriptions consistent with strategy use Credibility (CEQ)↑ ^Experimental v control^
2. Bowen and Marlatt (2009)	123	Experimental: mindfulness (urge surfing). Audio instructions: 11 min including cue reactivity Active control: self-selected coping (to use usual coping). Audio instruction: 11 min including cue reactivity	Age: 20.33 Male: 73.2% FTND: 2.31 Cigs/day: 5.33 Abstinence: 17.20 h	No details about instructions on strategy use during FU 24 h (94.30%) 7 days (90.20%)	Compared to self-guided task Number of cigarettes ↓ ^7 days^	Compared to self-guided task Craving (QSU brief) *↔* ^In session, 24 h, 7 days^ Negative affect (PANAS) *↔* ^In session, 24 h, 7 days^	None
3. Cropley et al. (2007)	30	Experimental: mindfulness (body scan). Audio instructions: 10 min Inactive control: control audio: 10-min audio	Age: 25.45 Male: 60.0% FTND: 4.75 Cigs/day: 18.00 Abstinence: 12.8 h ‘overnight’	N/A (in-session testing only)	None	Compared to inactive Craving (single item ‘desire to smoke’) ↓ Negative affect (withdrawal-related; MPSS): irritable ↔, tense ↔, restless ↔	None
4. Litvin et al. (2012)	162	Experimental: acceptance. Slide show: 10 min Suppression control Slide show: 10 min Inactive control: reading Slide show: 10 min	Age: 36.84 Male: 50.0% FTND: 5.33 Cigs/day: 20.10 Abstinence: 3.0 h	Explicit instructions to continue to use assigned strategy 3 days (69.73%)	Compared to suppression Latency to smoke *↔* ^Post-session^ Number of cigarettes *↔* ^3-day FU^ Compared to reading Latency to smoke *↔* ^Post-session^ Number of cigarettes *↔* ^3-day FU^	Compared to suppression Craving (one-item urge, ME, QSU-4) *↔* ^In session^ Negative affect (MF) *↔* ^In session^ Thoughts about smoking ↑ ^In session^ (suppression = reported fewer smoking thoughts) Compared to reading Craving (one-item urge, ME) ↓ ^In session^ Craving (QSU-4) *↔* ^In session^ Negative affect (MF) ↓ ^In session^ Thoughts about smoking *↔* ^In session^	Self-ratings of ‘suppression’ and ‘acceptance’ consistent with assigned strategy
5. May et al. (2011)	27	Active: mindfulness (body scan). Audio instructions: 10 min Inactive: mind wandering. Audio instructions: 10 min (NB: within subjects)	Age: 30.00 Male: 40.74% FTND: N/R Cigs/day: N/R Abstinence: 2.0 h	N/A (in-session testing only)	None	Compared to mind wandering Craving (factor 1 of the QSU) ↓ Smoking thought frequency (thought probes) ↓	None
6. Nosen and Woody (2013)	122	Experimental: mindfulness psycho-education. Slide show: 60–90 min Active control: standard smoking psycho-education. Slide show: 60–90 min Inactive control: no task. Filler questionnaires: 60–90 min	Age: 41.47 Male: 64.77% CDS: 48.55 Cigs/day: 16.49 Abstinence: N/R	No details about use of strategies during the FU period 24 h (89.0%) 4 days (N/R)	Compared to standard psycho-education Appraisal of Craving Questionnaire (ACQ)—strength of craving beliefs ↓^In-session^ Compared to no task Appraisal of Craving Questionnaire (ACQ)—strength of craving beliefs ↓^In-session^	Compared to standard psycho-education Craving (single-item VAS and QSU brief) *↔* in the morning; ↓ ^24-h FU^ in the evening (quitters only) Compared no task Craving (VAS single item) ↓^24-h FU^ in the morning and evening (quitters only) Craving (QSU brief) ↔ ^In-session^	None
7. Rogojanski et al. (2011)	61	Experimental: mindfulness (urge surfing). Audio instructions: duration not reported Suppression control: audio instructions: duration not reported	Age: 40.34 Male: 59.00% FTND: 4.57 Cigs/ day: 16.42 Abstinence: 0.8 h	Participants *not* instructed to use strategy 7 days (80.33%)	Compared to suppression Number of cigarettes (TLFB) *↔* ^7-day FU^	Compared to suppression Craving (VAS) *↔* ^In session, 7-day FU^ Negative affect (PANAS) ↓^7-day FU^	Credibility (CEQ) *↔*
8. Ruscio et al. (2015)	44	Experimental: mindfulness. Audio instructions: 20 min Inactive control: sham meditation. Audio instructions: 20 min	Age: 44.75 Male: 47.5% WISDM: 55.72 Cigs/day: 15.93 Abstinence: N/A (instructed to smoke as little or as much as you like during study)	Instructions to continue using assigned strategy (20 min/day) 7 days (72.7%) 14 days (72.7%; linear mixed model analysis)	Compared to sham meditation Number of cigarettes ↓^14-day FU^ (NB groups differed in cigarettes/day by 4.15, *p* = 0.06)	Compared to sham meditation Craving (single-item scale) ↔^14-day FU^ Negative affect (PANAS-NA) ↔^14-day FU^	None
9. Szasz et al. (2012)	94	Experimental: acceptance. Written instructions: 72 words Active control: reappraisal. Written instructions: 75 words Suppression control: written instructions: 66 words	Age: 23.02 Male:11.7% FTND: 3.14 Cigs/day: 18.62 Abstinence (mode): 1.88 h	N/A (in-session testing only)	None	Compared to reappraisal Craving (QSU brief) *↑* Negative affect (PANAS) *↑* Compared to suppression Craving (QSU brief) *↔* Negative affect (PANAS) *↔*	‘Adjusting,’ ‘concealing’ and ‘tolerating’ scores consistent with strategy use
10. Ussher et al. (2006)	60	Experimental: mindfulness (body scan). Audio instructions: 5 min Active control: isometric exercise. Audio instructions: 5 min Inactive control (passive sitting): 5 min	Age: 32.20 Male: 55.0% FTND: 3.92 Cigs/day: 18.83 Abstinence: 17.31 h	N/A (in-session testing only)	None	Compared to isometric exercise Craving (single item desire to smoke) *↔* Negative affect (withdrawal-related; MPSS): irritable *↑*; tense *↑*; restless *↑*, stressed *↑*, poor concentration ↔; depressed ↔ Compared to passive sitting Craving (single item desire to smoke) *↔* Negative affect (withdrawal-related; MPSS): irritable ↔; tense ↔; restless *↑*; stressed ↔; poor concentration ↔; depressed ↔	None
11. Ussher et al. (2009)	48	Experimental: mindfulness (body scan). Audio instructions: 10 min Active control: isometric exercise. Audio instructions: 10 min Inactive control: reading control: 10 min	Age: 27.80 Male: 64.6% FTND: 5.00 Cigs/day: 15.5 Abstinence: 16.7 h	N/A (in-session testing only)	None	Compared to isometric exercise Craving (single item desire to smoke) *↔* Negative affect withdrawal-related; MPSS: irritable ↔; tense ↔; restless ↔; stressed ↔; poor concentration ↔ Compared to reading Craving (single item desire to smoke) ↓ Negative affect (withdrawal-related; MPSS): irritable ↓; tense ↔; restless ↓; stressed ↓; poor concentration ↓	Credibility (CEQ) *↔* ^Experimental v Active^
12. Westbrook et al. (2013)	54	Experimental: mindfulness/acceptance. Instructions provided by the experimenter: 366 words Inactive control: look naturally. Instructions provided by the experimenter: 31 words (NB: within subjects)	Age: 45 Male: 69% FTND: 5.03 Cigs/day: 17.58 Abstinence: 12 h	N/A (in-session testing only)	None	Compared to looking neutrally Craving (single item) ↓ Negative affect (single item) ↓	None

*N*/*R* not reported, *N*/*A* not applicable or not assessed, *FU* follow-up, ↑ higher levels or higher scores on outcome measure relative to comparator within session and/or at FU (higher levels in the experimental condition signifying *lower* efficacy of the experimental condition, i.e. control conditions performed better), *↔* no difference between groups within session and/or at FU, *↓* lower levels or lower scores on outcome relative to comparators (lower levels generally signifying *superior* effect of experimental condition within session and/or FU), *PANAS* Positive and Negative Affect Schedule, *QSU* Questionnaire of Smoking Urges, *MPSS* Mood and Physical Symptoms Scale, *MF* the mood form, *VAS* visual-analogue scale single item, *ACQ* Appraisals of Craving Questionnaire, *CDS* Cigarette Dependence Scale, *FTND* Fagerström Test for Nicotine Dependence, *TLFB* Timeline Follow-Back, *CEQ* Credibility/Expectancy Questionnaire, *WISDM* Wisconsin Inventory of Smoking Dependence Motives

All but one study (May et al. 2011) reported the number of cigarettes per day, with a mean across studies of 15.98 cigarettes/day (see Table 1 for range). However, with the exception of Rogojanski et al. (2011) and Beadman et al. (2015) who used a validated procedure for assessing smoking behaviour (Timeline Follow-Back (TLFB); Brown et al. 1998), the method for assessing daily smoking was generally not well specified.

A period of abstinence prior to participation was a common requirement across the studies, with an average of 8.93 h (median 8.81 h) since last cigarette. This suggests that in general, strategies were applied in the context of significant levels of withdrawal-related craving (on top of cue-induced craving in some cases; see below). One study (Rogojanski et al. 2011) attempted to overcome potential ceiling levels of craving at baseline by instructing participants to smoke a cigarette 30 min prior to the experimental session. However, the downside of this approach is that the resulting satiety can obscure episodic craving effects. Indeed, these authors did not detect the expected increase in craving following their cue reactivity procedure, complicating the interpretation of effects of PFM strategy (urge surfing) on cue-induced craving.

Carbon monoxide (CO) levels were assessed in all studies except Bowen and Marlatt (2009) and Szasz et al. (2012). Four studies specified inclusion criteria based on CO levels (≥8: Litvin et al. 2012 and Nosen and Woody 2013; ≥10: Ruscio et al. 2016; ≥15 ppm: Ussher et al. 2009). Six studies also used CO levels to determine compliance with abstinence instructions prior to the experimental session (Cropley et al. 2007; Nosen and Woody 2013; Ruscio et al. 2016; Ussher et al. 2009; Ussher et al. 2006; Westbrook et al. 2013) and three assessed CO levels but did not report levels or clearly specify cutoff levels for participant inclusion (Beadman et al. 2015; May et al. 2011; Rogojanski et al. 2011). Except for Ruscio et al. (2016), who also assessed cotinine levels, no other biological assay of nicotine/smoking was used in the reviewed studies.

All of the studies examined sample characteristics at baseline in order to explore any pre-existing differences between the groups. Two (Beadman et al. 2015; Ussher et al. 2006) found small, random differences, one of which attempted to statistically determine the effect of these differences on the main outcomes (Beadman et al. 2015).

### Methodological Overview of Studies

Table 2 presents the key characteristics of the 12 studies. All studies primarily examined affective and cognitive outcomes acutely (within-session), although six (Beadman et al. 2015; Bowen and Marlatt 2009; Litvin et al. 2012; Nosen and Woody 2013; Rogojanski et al. 2011; Ruscio et al. 2016) assessed smoking behaviour, craving and/or affect during follow-up periods of up to 2 weeks.

**Table 2:** Methodological evaluation of studies

Study	Q1	Q2	Q3					Q4	Q5	Q6	Q7	Q8	Q9	Q10	Q11	Q12	Q13	Q14
			A	B	C	D	E											
Beadman et al. (2015)	N	Y	Y	Y	Y	Y	Y	Y	Y	Y	N	Y	Y	Y	Y	N	Y	Y
Bowen and Marlatt (2009)	N	Y	Y	Y	Y	Y	Y	N	Y	N	N	Y	Y	N	N	N	Y	Y
Cropley et al. (2007)	N	Y	Y	N	Y	N	Y	N	Y	Y	N	N/A	Y	N/A	N	N	N	Y
Litvin et al. (2012)	N	Y	Y	Y	N	Y	Y	N	Y	N	N	Y	Y	N	Y	Y	Y	Y
May et al. (2011)	N	N/A	Y	Y	Y	N	Y	N	Y	N	N	N/A	Y	N/A	N	N	Y	Y
Nosen and Woody (2013)	N	Y	–	–	–	Y	Y	N	Y	N	N	N/A	Y	N/A	N	Y	Y	Y
Rogojanski et al. (2011)	N	Y	Y	Y	Y	Y	Y	N	Y	N	N	N/A	Y	N	Y	N	Y	Y
Ruscio et al. (2016)	N	Y	Y	Y	Y	Y	Y	N	Y	Y	N	Y	Y	Y	N	N	N	Y
Szasz et al. (2012)	N	Y	Y	Y	N	Y	Y	N	Y	N	N	N	Y	N	N	Y	Y	N
Ussher et al. (2006)	N	Y	N/A	Y	Y	Y	Y	N/A	Y	N/A	N	N/A	Y	N/A	N	N	N	Y
Ussher et al. (2009)	N	Y	Y	Y	Y	Y	Y	N	Y	Y	N	N/A	Y	N/A	Y	N	N	Y
Westbrook et al. (2013)	N	N/A	N	Y	N	N	Y	N	Y	N	N	Y	Y	N	N	N	Y	Y

See text for details on the individual methodological study features summarised here

*Q1* blinding of assessor, *Q2* experimental conditions homogeneous, *Q3* strategies matched, *A* length, *B* readability, *C* key words, *D* engagement with material, *E* delivery method, *Q4* criterion 3 supported by independent raters, *Q5* strategy relevant to experimental challenge, *Q6* themes/quality of strategy supported by independent raters, *Q7* verbal summary of understanding of strategy, *Q8* reminder of strategy prior to physical/psychological challenge, *Q9* standardised instructions, *Q10* verbal summary of application of strategy, *Q11* credibility checks, *Q12* standardised manipulation check, *Q13* power calculation for group design, *Q14* experiential elements, *Y* present, *N* not present, *N*/*A* not applicable/not assessed

The majority of studies examined strategies that appeared to involve some form of mindfulness. Urge surfing was used in two studies (Bowen and Marlatt 2009; Rogojanski et al. 2011), the body scan in four (Cropley et al. 2007; May et al. 2011; Ussher et al. 2009; Ussher et al. 2006) and general mindfulness instructions in three (Nosen and Woody 2013; Ruscio et al. 2016; Westbrook et al. 2013). The term ‘general mindfulness’ is used here to describe instructions that included (Westbrook et al. 2013) or appeared to include (Nosen and Woody 2013) various instructions intended to increase mindful responding (bringing attention to, accepting, noticing, observing, not attempting to change sensations relevant to craving) that were not described as body scan or urge surfing. Alternatively, it refers to the use of various individual mindfulness exercises (urge surfing plus mindfulness of the breath, body, thoughts and emotions; Ruscio et al. 2016).

The specific effects of acceptance instructions were examined in two studies (Litvin et al. 2012; Szasz et al. 2012), and one study examined defusion (Beadman et al. 2015). No study specifically examined strategies involving self-as-context, values or committed action.

Regarding the control groups, six studies (Bowen and Marlatt 2009; Cropley et al. 2007; May et al. 2011; Rogojanski et al. 2011; Ruscio et al. 2016; Westbrook et al. 2013) employed one comparison condition. Of these, four (Cropley et al. 2007; May et al. 2011; Ruscio et al. 2016; Westbrook et al. 2013) used inactive controls (e.g. reading a neutral article), one used an active control (instructions to use usual coping strategies; Bowen and Marlatt 2009) and one (Rogojanski et al. 2011) used suppression. The remaining six studies employed two comparison conditions; three (Nosen and Woody 2013; Ussher et al. 2009; Ussher et al. 2006) used inactive and active controls (e.g. isometric exercise or standard smoking psycho-education about risk factors, health impact and strategies such as hypnosis, nicotine replacement aids and building social support), one (Litvin et al. 2012) used an inactive control and suppression and two (Beadman et al. 2015; Szasz et al. 2012) used suppression and an active control (reappraisal).

Eight studies (Beadman et al. 2015; Bowen and Marlatt 2009; Litvin et al. 2012; Nosen and Woody 2013; Rogojanski et al. 2011; Ruscio et al. 2016; Szasz et al. 2012; Westbrook et al. 2013) designed their strategies to be specific to craving and smoking, and most of these used exposure to in vivo cues to trigger cravings as part of the experimental procedure (Beadman et al. 2015; Bowen and Marlatt 2009; Litvin et al. 2012; Nosen and Woody 2013; Rogojanski et al. 2011; Szasz et al. 2012; Westbrook et al. 2013). May et al. (2011) aimed to implicitly induce craving by removing participants’ cigarettes at the beginning of the experiment. The remaining three studies (Cropley et al. 2007; Ussher et al. 2009; Ussher et al. 2006) employed strategies that did not specifically target craving and instead involved general body scanning, testing the effects of this strategy on background (withdrawal-related) craving.

### Specific Methodological Features of Studies

#### Strategy Instructions

Standardised strategy instructions were presented in verbal/audio or written form in all studies. However, no study used procedures for blinding experimenters to strategy allocation.

#### Strategy Integrity

The duration of strategy instructions between conditions was generally well-matched (Table 1). In three studies, strategy instructions were judged not to be matched with regards to the use of smoking-relevant words (Litvin et al. 2012; Szasz et al. 2012; Westbrook et al. 2013) and there was considerable heterogeneity between studies in the complexity of instructions and level of engagement required between conditions, with active/suppression controls generally well-matched to the PFM strategies and the inactive controls moderately well or inadequately matched. Two studies (Beadman et al. 2015; Ruscio et al. 2016) obtained independent checks on the theme/quality of the strategies and their relevance to the experimental challenge. Credibility was assessed in four studies (Beadman et al. 2015; Litvin et al. 2012; Rogojanski et al. 2011; Ussher et al. 2009).

Most studies targeted relevant addiction-related subjective experiences (e.g. acceptance of smoking*-*related thoughts or mindful responding to cigarette craving; Beadman et al. 2015; Bowen and Marlatt 2009; Litvin et al. 2012; Nosen and Woody 2013; Rogojanski et al. 2011; Ruscio et al. 2016; Szasz et al. 2012; Westbrook et al. 2013). Others encouraged generic awareness and acceptance of internal experiences (Cropley et al. 2007; May et al. 2011; Ussher et al. 2009; Ussher et al. 2006).

#### Manipulation Checks

As indicated in Table 2, standardised manipulation checks were rare (Litvin et al. 2012; Nosen and Woody 2013; Szasz et al. 2012) and no study performed checks on comprehension prior to participants employing the strategy. Additionally, amongst the studies that involved a psychological/physical challenge (Beadman et al. 2015; Bowen and Marlatt 2009; Litvin et al. 2012; Rogojanski et al. 2011; Szasz et al. 2012; Westbrook et al. 2013), only one (Beadman et al. 2015) assessed compliance with strategy instructions by retrospectively eliciting information from participants on strategy use.

### Outcomes

With the exception of Westbrook et al. (2013) and Ruscio et al. (2016), all studies relied primarily on self-reporting of smoking-related outcomes. In contrast, Westbrook et al. (2013) was primarily a neuroimaging study, with additional self-report assessment (which are the subject of this review), and Ruscio et al. (2016) supplemented self-report data with objective biological assays of smoking.

#### PFM-Relevant Outcomes: Smoking Behaviour and Smoking-Related Meta-cognition

Five of the 12 studies assessed smoking behaviour (Tables 1 and 2). This involved assessment of behaviour that occurred outside of the experimental session over extended periods relative to the duration of the strategy (Beadman et al. 2015; Bowen and Marlatt 2009; Litvin et al. 2012; Rogojanski et al. 2011; Ruscio et al. 2016). Changes in smoking at follow-up were assessed using self-report, but only Ruscio et al. (2016) attempted to verify behavioural changes biochemically (carbon monoxide and cotinine levels). Rogojanski et al. (2011) reported a significant reduction in nicotine dependence in the PFM strategy group, as measured with the FTND. However, it seems unlikely that this reflected a true reduction in dependence given that the behaviours assessed by the FTND are unlikely to change reliably during a short follow-up period. Indeed, the authors report low levels of reliability of this scale in their sample. Moreover, they found no corresponding reduction in TLFB smoking levels.

Three of the five studies that examined self-reported smoking behaviour during follow-up (7–14 days) reported significant reductions following the PFM strategy relative to a suppression (Beadman et al. 2015), active (Bowen and Marlatt 2009) or inactive control (Ruscio et al. 2016). However, when mean ESs were examined, there was no significantly superior effect of PFM strategies relative to suppression (Beadman et al. 2015; Litvin et al. 2012; Rogojanski et al. 2011; *k* = 3, *n* = 171; ES = −0.25, 95% CI [−0.58, 0.08], *z* = 1.47, *p* = 0.14), active controls (Beadman et al. 2015; Bowen and Marlatt 2009; *k* = 2, *n* = 150; ES = −0.25, 95% CI [−0.57, 0.07], *z* = 1.53, *p* = 0.13) or inactive controls (Litvin et al. 2012; Ruscio et al. 2016; *k* = 2, *n* = 111; ES = −0.04, 95% CI [−0.52, 0.43], *z* = 0.18, *p* = 0.85). Given the small number of studies in each of these meta-analyses, however, these ESs should be considered provisional.

Missing data was present in all studies that examined smoking behaviour across days, with retention varying considerably (Table 1). Three of these mentioned how missing data was handled and/or used statistical methods for minimising the resulting bias. One study employed list-wise deletion in the reported analysis, a potentially problematic approach given the amount of missing data (~30%; Litvin et al. 2012). Similar levels of missing data were addressed using mixed-effect modelling in one study (Ruscio et al. 2016) and multiple imputation in another (Beadman et al. 2015).

Two studies examined meta-cognitive/affective outcomes relevant to smoking (Beadman et al. 2015; Nosen and Woody 2013) that were considered to constitute PFM-consistent outcomes (Table 1). Both showed effects favouring the PFM strategy compared to inactive (Nosen and Woody 2013) or suppression controls (Beadman et al. 2015). Interestingly, Beadman et al. (2015) found that the PFM and active control groups (reappraisal) showed similar reductions in smoking-related experiential avoidance, but only the PFM strategy group’s levels of experiential avoidance were *significantly* lower than the suppression control (Beadman et al. 2015).

#### Effects on Frequency and Intensity of Internal Subjective Experience

All reviewed studies assessed craving (or urge/desire to smoke). Six of the seven studies that compared a PFM strategy with an inactive control condition reported significantly larger reductions in cravings in the PFM group (Cropley et al. 2007; Litvin et al. 2012; May et al. 2011; Nosen and Woody 2013; Ussher et al. 2009; Westbrook et al. 2013), although these effects were generally most evident immediately after strategy use (Cropley et al. 2007; Litvin et al. 2012; May et al. 2011; Ussher et al. 2009; Westbrook et al. 2013). The overall mean ES from available data (Litvin et al. 2012; May et al. 2011; Ruscio et al. 2016; Ussher et al. 2009; Ussher et al. 2006; Westbrook et al. 2013) indicated that PFM-based strategies were superior at reducing craving relative to inactive control (*k* = 6, *n* = 386; ES = −0.24, 95% CI [−0.44, −0.03], *z* = 2.30, *p* = 0.02). Of the four studies that used a suppression condition (Beadman et al. 2015; Litvin et al. 2012; Rogojanski et al. 2011; Szasz et al. 2012), none reported any difference in craving compared to the PFM strategy conditions and the overall ES was not significant (*k* = 3, *n* = 217; ES = −0.08, 95% CI [−0.38, 0.22], *z* = 0.52, *p* = 0.61, based on data from Beadman et al. (2015), Litvin et al. (2012) and Rogojanski et al. (2011)). Comparisons to active control conditions on the other hand were more mixed (Beadman et al. 2015; Bowen and Marlatt 2009; Nosen and Woody 2013; Szasz et al. 2012; Ussher et al. 2009; Ussher et al. 2006; note that an insufficient proportion of the data was available to determine an ES). Four studies reported similar changes in craving in the PFM strategy and active control conditions (Beadman et al. 2015; Bowen and Marlatt 2009; Ussher et al. 2009; Ussher et al. 2006). Of note, whilst Beadman et al. (2015) did not find any difference between active control (reappraisal) and PFM strategy (defusion) on acute craving, the reappraisal group, but not the PFM strategy group, demonstrated a reduction in craving relative to suppression. This effect was evident immediately and 24 h after in-session instructions. As noted above, the same study reported a reduction in experiential avoidance following defusion (but not after reappraisal), a dissociation of effects that are in line with both the process model of emotion regulation and PFM theory (Beadman et al. 2015). Two of the remaining three studies reported opposing effects, either an advantage for the PFM strategy (but only amongst those who made a quit attempt and only for evening craving levels at follow-up; Nosen and Woody 2013) or an advantage for the active (reappraisal) strategy relative to the PFM strategy (acceptance; Szasz et al. 2012). Indeed, the latter study showed that participants experienced increased craving after the PFM and suppression control strategies, but not after the active control (reappraisal) strategy.

The other main measure of internal experience was negative affect, including withdrawal-related negative affect, assessed in 10 studies (Beadman et al. 2015; Bowen and Marlatt 2009; Cropley et al. 2007; Litvin et al. 2012; Rogojanski et al. 2011; Ruscio et al. 2016; Szasz et al. 2012; Ussher et al. 2009; Ussher et al. 2006; Westbrook et al. 2013). Of the six studies comparing the effects of a PFM strategy to an inactive control (Cropley et al. 2007; Litvin et al. 2012; Ruscio et al. 2016; Ussher et al. 2009; Ussher et al. 2006; Westbrook et al. 2013), three reported a larger within-session reduction in negative affect in the PFM group (Litvin et al. 2012; Ussher et al. 2009; Westbrook et al. 2013). The overall ES for this comparison, based on data from five of the six studies (Litvin et al. 2012; Ruscio et al. 2016; Ussher et al. 2009; Ussher et al. 2006; Westbrook et al. 2013), was significant and favoured the PFM strategy (*k* = 5, *n* = 332; ES = −0.42, 95% *CI* [−0.65, −0.19], *z* = 3.62, *p* < 0.01). Amongst the four studies that compared a PFM strategy to suppression (Beadman et al. 2015; Litvin et al. 2012; Rogojanski et al. 2011; Szasz et al. 2012), one reported larger reductions in negative affect immediately after strategy use and also after a long delay in the PFM group (7-day follow-up; Rogojanski et al. 2011). In studies comparing PFM strategies to active controls (Beadman et al. 2015; Bowen and Marlatt 2009; Szasz et al. 2012; Ussher et al. 2009; Ussher et al. 2006), three reported no difference to the PFM strategy (Beadman et al. 2015; Bowen and Marlatt 2009; Ussher et al. 2009), but two reported a more favourable outcome in the active control relative to the PFM group (Szasz et al. 2012; Ussher et al. 2006). Insufficient data was available to report an overall mean ES.

The final aspect of internal experience assessed in two of the reviewed studies was frequency of smoking thoughts. In Litvin et al.’s (2012) study, suppression resulted in an acute reduction in smoking thoughts, which were significantly less frequent than in the PFM strategy group. To the extent that smoking-related thoughts contribute to craving (May et al. 2011), the latter findings suggest a more favourable outcome for suppression although both emotion regulation theory and the PFM would predict that any immediate reductions in thought frequency after suppression would be followed by rebound effects. However, Litvin and colleagues’ study (Litvin et al. 2012) did not find evidence of such delayed effects. In contrast, May et al. (2011) showed that compared to an inactive (mind wandering) control, the PFM strategy (body scan) resulted in a reduction in the frequency of smoking thoughts.

To summarise these outcomes, the most consistent findings across studies were immediate reductions in craving and negative affect when a PFM strategy was compared to an inactive control. However, when the PFM strategy was compared to more stringent control groups, there was no consistent evidence of differences between conditions, either in relation to craving/negative affect or PFM outcomes. However, it should be noted that with the exception of Beadman et al. (2015), no study provided explicit differential hypotheses regarding the effects of PFM versus active control strategies on behaviour and craving. As such, it was generally not clear whether study authors were testing for superiority or equivalence/non-inferiority of the PFM strategy relative to active control conditions.

## Discussion

In this review, we provide a systematic synthesis of findings from, and methodological characteristics of, studies examining the isolated effects of PFM strategies that are used as therapeutic ingredients in PFM interventions for smoking cessation. Where sufficient data were (made) available, ESs were reported, although ESs for PFM outcomes were based on a small number of studies and therefore lacked the precision to make a robust determination of strategy effects. As it stands, the qualitative and limited quantitative findings did not indicate beneficial effects of specific, brief and isolated PFM strategies for smoking *behaviour*. In contrast, PFM strategy use was associated with reductions in acute craving and negative affect when compared to inactive controls, but not active strategies, which is the more stringent comparison.

Before proceeding, it is worth reemphasising that the purpose of controlled experimental laboratory component studies is rarely to establish clinical efficacy, but rather to develop insights about the validity of theoretical and psychotherapeutic concepts that can then be used to refine theory or streamline treatment (e.g. by identifying potentially inert or weakly effective components; Hayes et al. 2011). To be informative in this regard, clear theoretical foundations and high levels of internal validity should be brought to bear. However, all of the studies reviewed here have some limitations in this respect, which currently leave some uncertainty about their true impact on emotion regulation and PFM-specific outcomes.

Amongst the most significant limitations of the reviewed studies was a lack of theoretically driven predictions relating to the effects of PFM strategies (i.e. predictions that were clearly grounded in the PFM/mindfulness theory) and limited use of appropriate (PFM-consistent) assessment measures. Some of the reviewed studies seemed, either implicitly or explicitly, to consider the PFM strategies to be *emotion regulation* techniques, that is, strategies geared towards controlling (reducing) the occurrence or intensity of unwanted or maladaptive internal experiences. For example, one study stated that according to ACT theory, a larger reduction in negative affect and craving following acceptance instructions was expected relative to comparison conditions including reappraisal (Szasz et al. 2012). However, this prediction is not, in fact, consistent with PFM theory. Another study, whilst providing explicit differential hypotheses for reappraisal and defusion, based on emotion regulation and ACT theories, respectively, nonetheless described both as emotion regulation strategies (Beadman et al. 2015). These conceptual issues can have consequences for decisions on study design.

For example, the tendency to conceptualise PFM techniques as emotion regulation strategies may partly explain why only six studies examined outcomes more suited to testing PFM strategy effects, but *all* examined negative affect and/or craving. Several of these provided specific hypotheses (although the theoretical bases for these hypotheses were generally unclear) in relation to the latter outcomes, generally predicting and reporting acute reductions in intensity of craving in the PFM strategy groups. Yet, unlike emotion regulation-based (cognitive-behavioural) strategies, which assume that modification of maladaptive thoughts precedes a relatively immediate reduction in the intensity and frequency of feeling states, *abrupt* reductions in craving after very brief PFM strategy use were less expected. Indeed, it is equally plausible that enhanced interoceptive attention via mindfulness or acceptance instructions would temporarily *exacerbate* craving and negative mood, particularly when limited explanation of, or experiential practice with, the relevant techniques is provided. However, given that the reviewed studies did tend to show reductions in intensity of subjective experiences, we consider a number of potential explanations for apparent acute emotion regulatory effects of PFM strategies.

Firstly, recent efforts to integrate aspects of the PFM (mindfulness in particular) into the process model of emotion regulation (Farb et al. 2014) and conceptualise these as unique forms of early-stage attentional regulation may provide testable, mechanistic proposals for “how attention deployment leads to *attenuation* of negative emotions” (Farb et al. 2014, p. 549). For example, mindfulness may feed into reappraisal processes, and enable *novel appraisals* of emotional experiences. Related to this, state mindfulness has been shown to mediate mindfulness training effects on reappraisal (Garland et al. 2015b). Secondly, extended simultaneous processing of multiple cues in the context of a widened scope of attention and approach orientation, in the absence of reinforcement, may reduce conditioned responding through extinction (Treanor 2011). One line of empirical support for this idea is the finding that reduced drug craving is associated with higher levels of home practice of mindfulness (potentially reflecting greater/more prolonged exposure to conditioned stimuli in a variety of contexts) following a course of treatment with MBRP (Grow et al. 2015). Thirdly, PFM strategies might disrupt the elaboration of smoking-related cognition (especially mental imagery)—which is implicated in craving—by competing for the limited cognitive resources that normally subserve such elaboration (May et al. 2015). Finally, reductions in craving and negative affect may simply reflect expectancy effects. Although perhaps the least scientifically appealing, it is difficult to discount this possibility since the mediating effect of expectancy was not generally tested in the reviewed studies, and the between-group differences were most clearly observed under conditions that controlled the least well for it (i.e. when an inactive control was used).

Objective assessments of cue reactivity/craving could help resolve concerns regarding expectancy effects. In this respect, further studies (e.g. Westbrook et al. 2013) examining the neural circuitry subserving eliminative (e.g. reduction in craving) and potentially generative (e.g. increased positive affect; Garland et al. 2015a) pathways of PFM strategies might be useful. Moreover, such studies would allow an exploration of how neuroplasticity mediates the effects of PFM strategies on behavioural change (McConnell and Froeliger 2015) and help us determine the degree to which the neural substrates of PFM strategy effects overlap with those of emotional regulation strategies, like reappraisal. More generally, future studies that focus on the mechanisms through which PFM strategies exert their effects (whether through changes in cognition or affect, or not) would be valuable in helping to test and/or refine theoretical predictions of the PFM and the interventions guided by this model.

In addition, participant expectancy effects could be minimised in future studies by blinding participants to study aims and ensuring that control conditions have similar levels of credibility and expectancy as the PFM condition. Indeed, Beadman et al. (2015) found that despite attempting to match instructions closely for experimental (defusion), active-control (reappraisal) and suppression-control conditions, participants rated the suppression condition as significantly less credible. As such, an untested assumption of equivalent credibility across experimental and control groups cannot be considered safe. Moreover, no study has yet attempted to limit *experimenter* expectancy through blinding. This could be relatively easily achieved through automated experimental procedures and would significantly allay concerns about experimenter allegiance.

Related to issues of credibility and expectancy, the fact that PFM-guided interventions do not primarily aim to reduce symptom intensity may be experienced as counterintuitive by participants (Eifert and Forsyth 2005). As such, an assessment of participants’ understanding of strategy instructions seems crucial, yet only two of the reviewed studies assessed understanding directly (Litvin et al. 2012; Nosen and Woody 2013). Of these, Litvin et al. (2012) reported poorer understanding of the PFM strategy compared to control instructions. ACT commonly uses metaphors, stories and experiential exercises to illustrate unfamiliar and abstract ideas. Use of such exercises *prior to* participants employing the PFM strategy would be an important refinement for future studies, although assessment of comprehension and compliance would still be essential.

In relation to the relatively infrequent use of smoking behaviour as an outcome in the reviewed studies, one potential concern might have been that a measurable reduction in smoking after very brief, single-session instructions was deemed unlikely. Indeed, significant reductions in the number of cigarettes smoked over an extended (several days or weeks) follow-up seem a particularly stringent test of efficacy of PFM strategies. Yet, two of the reviewed studies did indeed show reductions in smoking in the PFM strategy group (Bowen and Marlatt 2009; Ruscio et al. 2016). Of these, the reductions in number of cigarettes per day observed by Bowen and Marlatt (2009) at 7-day follow-up are particularly striking. Their within-session strategy instructions were only 11 min in duration, and participants did not appear to be provided with explicit instructions to practice the PFM strategy (urge surfing) during the follow-up period. This suggests that even extremely brief strategy instructions can have a measurable effect on (self-reported) smoking. However, a number of studies did not include a follow-up assessment period during which smoking was monitored. In these circumstances, alternative approaches to examining smoking behaviour could still be used, and employed within the same approximate time frame as the craving and negative affect assessments. For example, latency to the first cigarette after the experimental session could be assessed (remotely) or a laboratory analogue of relapse could be used (Froeliger et al. 2017). In addition, smoking topography (i.e. the frequency and duration of puffs) is also a reliable and valid index of changes in smoking behaviour (Lee et al. 2003). Researchers might therefore consider using these methods to assess smoking behaviour in future single-session experiments testing PFM strategies.

It should be noted that whilst our search terms included all aspects of the PFM, only studies on mindfulness, acceptance and defusion were identified. No appropriate studies of values or committed action based on PFM theory met the criteria, and as such, the effects of these individual intervention components remain unclear. It could be argued that self-affirmation studies should have been included in this review, since these often require participants to recall and reflect upon their values, which can have beneficial effects on drug/alcohol use (e.g. Kamboj et al. 2016). However, such studies—which typically assess the effects of affirmation on threatened ‘self-integrity’—are derived from social psychological theory developed independently of, and without reference to, the PFM or ACT. Future PFM-informed laboratory experiments focusing on values and committed action will be facilitated by recently developed assessment instruments that are firmly rooted in the PFM.

We must acknowledge some limitations of the current review. The focus of the review was primarily on methodological features of studies. Meta-analysis relies on open science practices, which are becoming increasingly prevalent. A greater availability of data would have strengthened this review by allowing a more comprehensive quantitative reporting to supplement the qualitative appraisal of reviewed studies. On the other hand, the number of available studies would still have been relatively small. As it stands, our findings relating to aggregate ESs were incomplete and potentially susceptible to bias.
